# Sequence Analysis of *In Vivo*-Expressed HIV-1 Spliced RNAs Reveals the Usage of New and Unusual Splice Sites by Viruses of Different Subtypes

**DOI:** 10.1371/journal.pone.0158525

**Published:** 2016-06-29

**Authors:** Yolanda Vega, Elena Delgado, Jorge de la Barrera, Cristina Carrera, Ángel Zaballos, Isabel Cuesta, Ana Mariño, Antonio Ocampo, Celia Miralles, Sonia Pérez-Castro, Hortensia Álvarez, Isabel López-Miragaya, Elena García-Bodas, Francisco Díez-Fuertes, Michael M. Thomson

**Affiliations:** 1 HIV Biology and Variability Unit, Centro Nacional de Microbiología, Instituto de Salud Carlos III. Majadahonda, Madrid, Spain; 2 Bioinformatics Unit, Centro Nacional de Microbiología, Instituto de Salud Carlos III. Majadahonda, Madrid, Spain; 3 Genomics Unit, Centro Nacional de Microbiología, Instituto de Salud Carlos III. Majadahonda, Madrid, Spain; 4 Hospital Arquitecto Marcide. Ferrol, A Coruña, Spain; 5 Complejo Hospitalario Universitario de Vigo. Vigo, Pontevedra, Spain; 6 AIDS Immunopathogenesis Unit. Centro Nacional de Microbiología, Instituto de Salud Carlos III. Majadahonda, Madrid, Spain; International Centre for Genetic Engineering and Biotechnology, ITALY

## Abstract

HIV-1 RNAs are generated through a complex splicing mechanism, resulting in a great diversity of transcripts, which are classified in three major categories: unspliced, singly spliced (SS), and doubly spliced (DS). Knowledge on HIV-1 RNA splicing *in vivo* and by non-subtype B viruses is scarce. Here we analyze HIV-1 RNA splice site usage in CD4^+^CD25^+^ lymphocytes from HIV-1-infected individuals through pyrosequencing. HIV-1 DS and SS RNAs were amplified by RT-PCR in 19 and 12 samples, respectively. 13,108 sequences from HIV-1 spliced RNAs, derived from viruses of five subtypes (A, B, C, F, G), were identified. In four samples, three of non-B subtypes, five 3’ splice sites (3’ss) mapping to unreported positions in the HIV-1 genome were identified. Two, designated A4i and A4j, were used in 22% and 25% of *rev* RNAs in two viruses of subtypes B and A, respectively. Given their close proximity (one or two nucleotides) to A4c and A4d, respectively, they could be viewed as variants of these sites. Three 3’ss, designated A7g, A7h, and A7i, located 20, 32, and 18 nucleotides downstream of A7, respectively, were identified in a subtype C (A7g, A7h) and a subtype G (A7i) viruses, each in around 2% of *nef* RNAs. The new splice sites or variants of splice sites were associated with the usual sequence features of 3’ss. Usage of unusual 3’ss A4d, A4e, A5a, A7a, and A7b was also detected. A4f, previously identified in two subtype C viruses, was preferentially used by *rev* RNAs of a subtype C virus. These results highlight the great diversity of *in vivo* splice site usage by HIV-1 RNAs. The fact that four of five newly identified splice sites or variants of splice sites were detected in non-subtype B viruses allows anticipating an even greater diversity of HIV-1 splice site usage than currently known.

## Introduction

**Fig 1:**
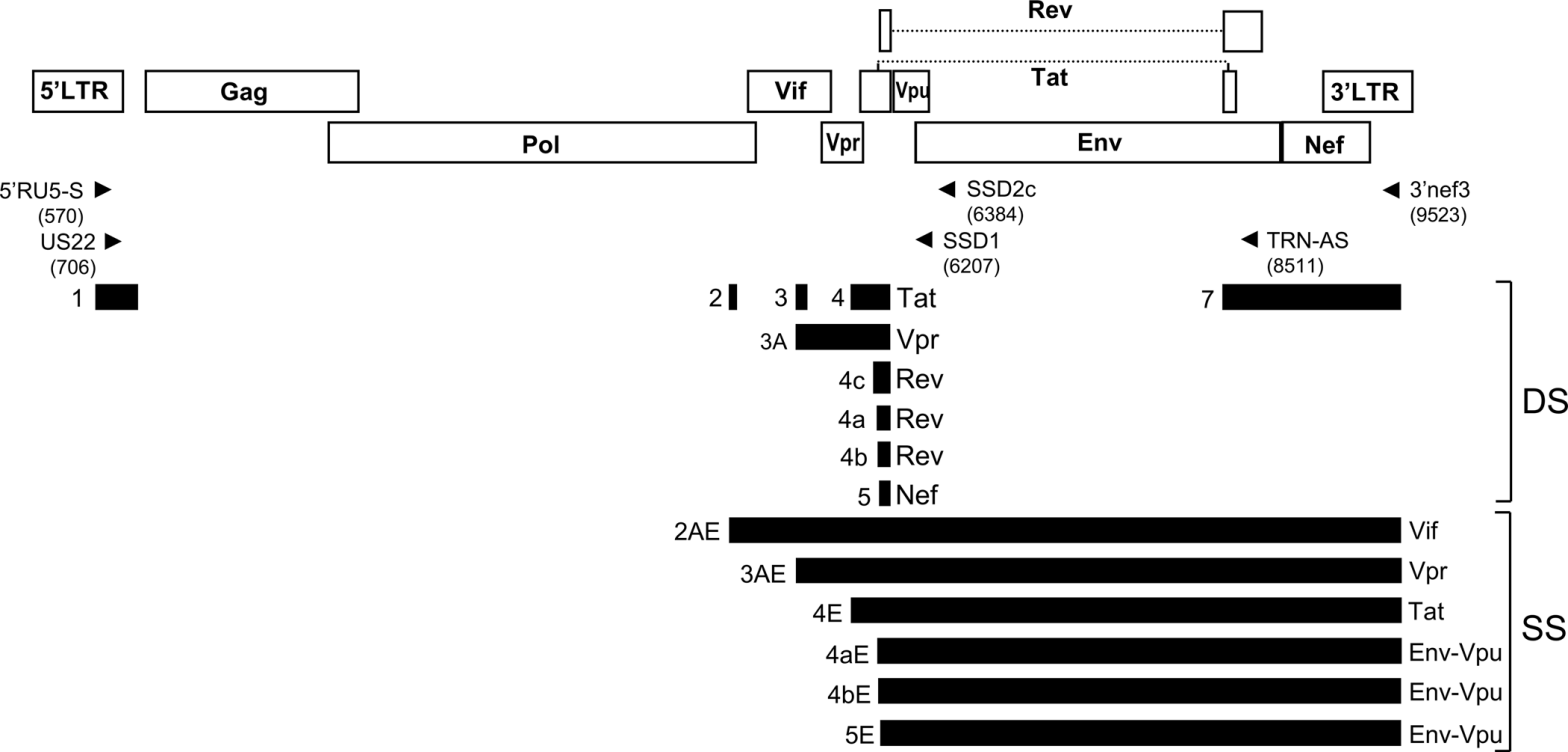
Schematic depiction of HIV-1 splicing and locations of PCR primers. Open reading frames are shown as open boxes and exons as black bars. Exons are named as previously [1,6]. All spliced transcripts incorporate exon 1 and all DS transcripts incorporate exon 7. Optionally, noncoding exons 2 or 3 or both can be incorporated into *nef*, *rev*, *tat*, or *env-vpu* transcripts, and exon 2 into *vpr* transcripts. A minority of *nef* RNAs are generated by splicing from exon 1 to exon 7. Proteins encoded in spliced RNAs are indicated on the right of the middle exon in DS RNAs and of the 3’-terminal exon in SS RNAs. Locations of sequences recognized by primers used for RT-PCR and nested PCR for DS and SS RNA amplification are indicated with arrows, with HXB2 positions of the primers’ 3’ ends in parentheses. 5’RU5-S and 3’nef3 were used for RT-PCR and US22 and TRN-AS for nested PCR for DS RNA amplification; 5’RU5-S and SSD2c were used for RT-PCR, and US22 and SSD1 for nested PCR amplification of SS RNAs.

HIV-1 RNAs are transcribed from a single promoter at the 5’ long terminal repeat and their relative expression is regulated through the alternative usage of splice sites. According to splicing events involved in their generation, HIV-1 transcripts are assigned to three major categories [1–6] (Fig 1): (1) unspliced RNA, coding for Gag-Pol and Pol polyproteins; (2) doubly spliced (DS) transcripts, generated by excision of major introns overlapping Gag-Pol and Vpu and Env open reading frames, coding for Tat, Rev, Nef, and Vpr proteins; and (3) singly spliced (SS) transcripts, generated by excision of the Gag-Pol intron, coding for Env, Vpu, Vif, Vpr, and a truncated Tat protein. The doubly or singly spliced RNA designations, employed here and in the literature, do not reflect the total number of splicing events but instead represent the number of major splicing events, with the exception of a singly spliced 1.7 Nef-encoding RNA, which is usually assigned to the DS category (Fig 1). A fourth category of short spliced RNAs, using 3’ splice sites (3’ ss) near the HIV-1 genome’s 3’ end, has been identified in two isolates *in vitro* and in a minority of viruses *in vivo* [7–9]; however, their frequency of expression, relative abundance, and function still remain to be defined. The complexity of HIV-1 splicing is further increased by several additional factors: (1) the usage of redundant closely spaced 3’ss for generation of *rev* RNAs, of which eight have been reported [1,6,9–13]; (2) the optional incorporation of small noncoding exons in the leader sequence (in addition to the 5’-terminal exon 1, common to all HIV-1 spliced RNAs): exons 2 or 3 or both in *tat*, *rev*, *nef*, and *env*-*vpu* RNAs, and exon 2 in *vpr* RNAs [1–6]; (3) the uncommon usage of other splice sites in some isolates [1,7–9,14–18].

The stability and nucleo-cytoplasmic transport of unspliced and SS transcripts requires the build-up of a certain threshold of Rev protein concentration. This determines that HIV-1 RNAs are expressed in a temporally-regulated fashion: DS transcripts, whose cytoplasmic expression is Rev-independent, are detected first, and, subsequently, after a certain concentration of Rev protein is reached, SS and unspliced RNAs are expressed [19,20].

Most HIV-1 RNA splice sites exhibit suboptimal efficiencies [21–25], which allow for the regulation of their relative usage through the action of cellular splicing regulatory factors binding to splice enhancer or suppressor elements in the HIV-1 genome [26]. The alterations in balanced splicing of HIV-1 transcripts may exert a strong negative effect on viral replication. These may be provoked by mutations in splice sites [6,27] or splice regulatory elements [28–30], or affecting RNA secondary structure at splice sites [31,32], or by manipulations in the expression of cellular splicing regulatory proteins [30,33].

The great majority of studies on HIV-1 RNA splicing have been performed with isolates of subtype B, which is predominant in Western countries. Published reports on non-subtype B viruses are limited to the study of two group O [11,34] and three subtype C [12] isolates. In these studies, RNA splice sites used by *rev* RNAs previously unreported in subtype B viruses were identified: A4e in a group O virus [11], A4f in two subtype C viruses, and A4g in one subtype C virus [12].

The majority of studies on HIV-1 splicing have been carried out in *in vitro* assays using a limited number of cell line-adapted isolates [1–6,9,16,35,36]. Most studies on *in vivo* expression of HIV-1 RNAs were limited to examining the expression of different transcript categories without analyzing individual transcripts within each category [36–48]. To our knowledge, only two studies analyzing *in vivo* expression of individual HIV-1 transcripts have been published, and in only one of them sequencing was used. In the first study [49], HIV-1 splicing patterns were analyzed in peripheral blood mononuclear cells (PBMCs) of HIV-1-infected individuals by reverse transcriptase polymerase chain reaction (RT-PCR) using a radiolabeled primer. Amplified products were identified according to their size through denaturing polyacrylamide gel electrophoresis. It was observed that the splicing patterns in each patient were conserved over the years and differed between individuals. Usage of splice sites was generally consistent with previous *in vitro* findings. The second study involved sequencing clones of RT-PCR products derived from DS and SS HIV-1 RNAs, amplified from PBMCs from 5 HIV-1-infected individuals, but only 94 clones were sequenced (10–31 per individual) [8].

In this study, we analyze *in vivo* HIV-1 splice site usage within the DS and SS categories through pyrosequencing using a greater number of clinical samples from HIV-1-infected individuals than in previous *in vivo* studies [8,49]. Since RNA splicing patterns may vary according to cell type [2,35,50] and activation state [51–55], a more homogeneous cell population was also used. For this, we isolated CD4^+^CD25^+^ lymphocytes, representing the activated T-lymphocyte population, obtained from 19 HIV-1-infected individuals at different stages of the infection. These cells were chosen because, among circulating cells, they represent the source of the great majority of HIV-1 virions [56–58]. The results allowed the detection of the *in vivo* usage of five new HIV-1 RNA splice sites, four of them in non-subtype B viruses, and several unusual ones.

## Materials and Methods

### Ethics statement

Written informed consent was obtained from all participants in the study, which was approved by the Bioethics and Animal Well-being Committee of Instituto de Salud Carlos III, Majadahonda, Madrid, Spain (Report # CEI PI 04_2010).

### Samples

Whole blood (20–50 ml) was collected from 22 HIV-1-infected antiretroviral drug-naïve individuals at diverse infection stages.

### CD4^+^CD25^+^ lymphocyte isolation

PBMCs were separated by density gradient centrifugation on Ficoll. CD4^+^CD25^+^ lymphocytes were obtained through immunomagnetic separation using, sequentially, CD4^+^ T Cell isolation Kit II, human, and CD25 MicroBeads II, human (Miltenyi Biotec, Bergisch Gladbach, Germany), following manufacturer’s instructions.

### RNA extraction and RT-PCR amplification of DS and SS HIV-1 RNAs

Total RNA was extracted using RNAeasy kit (Qiagen, Venlo, Netherlands) following manufacturer’s instructions. HIV-1 doubly and singly spliced RNAs were amplified in separate reactions by RT-PCR, followed by nested PCR, as previously described [12], with these modifications: (1) the antisense primer used for nested PCR of SS RNAs was SSD1 (CTCTCATTGCCACTGTCTTCTGCTC, HXB2 positions 6207–6231); (2) a different multiplex identifier (MID) sequence was appended to the 5’ of nested PCR primers used for each reaction, allowing for multiplexed analysis; and (3) 35 cycles were used for the nested PCR. RT-PCRs (35 cycles) were done with a mixture of SuperScript III Reverse Transcriptase (Life Technologies, Carlsbad, CA, USA), Biotaq DNA Polymerase (Bioline, London, UK), and Pfu DNA Polymerase (Stratagene, Cedar Crek, TX, USA), and nested PCRs with a mixture of Biotaq DNA Polymerase and Pfu DNA Polymerase. Locations in the HIV-1 genome of sequences recognized by primers are shown in Fig 1. PCR products were visualized by electrophoresis in 1% agarose gels and staining with SYBR Safe DNA Gel Stain (Life Technologies, Carlsbad, CA, USA). Due to budget limitations, only 12 samples were randomly selected for amplification and sequence analysis of SS RNAs among those with positive amplification for DS RNAs.

### Sequencing

Amplified cDNA products from different samples were quantified using Quant-iT PicoGreen dsDNA Assay Kit (Life Technologies, Carlsbad, CA, USA) and Agilent 2100 Bioanalyzer High Sensitivity DNA chips (Agilent Technologies, Santa Clara, CA, USA) and equimolarly pooled at concentration of 10^9^ molecules/μl prior to emulsion PCR. Enriched DNA beads were subjected to 400 cycles of pyrosequencing in GS Junior plus System (454 Life Sciences, Branford, CT, USA) using amplification mixes and PCR conditions recommended by the manufacturer for long amplicons. In the processing of raw reads, the most stringent analysis pipeline for long amplicons (Long Amplicons #1 pipeline, 454 Sequencing System Software Manual, v2.9, Part B, p. 27) was applied. Reads were deposited in the European Nucleotide Archive, with accession number PRJEB13105. Data associated with the submitted sequences are shown in supplementary S1 Table.

### Subtype assignation

HIV-1 subtype assignation was performed using the online program COMET (COntext-based Modeling for Expeditious Typing) [59] and with phylogenetic analyses with FastTree2 [60], applying the GTR+CAT evolutionary model. Phylogenetic analyses were done using the exon 5/exon 7 fragment, common to all DS RNAs, from 20 randomly chosen DS sequences from each sample, aligned with MAFFT (Multiple Alignment using Fast Fourier Transform) v.7.215 [61]. Trees were viewed with MEGA v.5.2 [62].

### Assignation of sequences to HIV-1 spliced transcripts

Sequences were assigned to known HIV-1 transcripts by mapping reads to reference sequences of HIV-1 transcripts using Burrows-Wheeler Aligner’s Smith-Waterman alignment (BWA-SW) [63]. HIV-1 reference sequences were generated by all possible combinations of mutually compatible exons of all reported HIV-1 exons of the identified subtypes, joined consecutively in the 5’-3’ order of their locations in the HIV-1 genome. The isolates used to generate reference sequences were HXB2 (subtype B), Q23_17 (subtype A), 96BW0502 (subtype C), 93BR020_1 (subtype F), and X558 (subtype G). BWA-SW parameters were adjusted empirically by comparing results to a set of manually aligned reads. Specifically, BWA-SW parameters were set to: mark multi-part alignments as secondary (-M); gap extension penalty set to 10 (-r10); maximum seeding interval set to 75 (-s 75); and Z-best set to 5 (-z 5). Sequences with ambiguous assignations with BWA-SW (i.e., those which were assigned to more than one transcript) were assigned to individual HIV-1 transcripts by alignment with reference sequences of HIV-1 spliced transcripts using MAFFT v.7.215 [61], parameterized to automatically select the appropriate strategy (—auto), with manual curation of alignments. Sequences that could not be assigned to reference transcript sequences by alignment with BWA-SW or MAFFT were mapped to the HIV-1 HXB2 reference genome using the online Sequence Locator program [64] to repeatedly align reads to the HXB2 genome, identify single exons, remove the identified exon sequence and realign the trimmed read to HXB2 until all identifiable exons were found. In all cases, identification of a new 3’ss required that the usual sequence elements of metazoan 3’ss (an adjacent AG dinucleotide and a nearby pyrimidine-rich tract) were present upstream of the putative 3’ss. Similarly, identification of a new 5’ splice site required that the highly conserved GT dinucleotide be present immediately downstream of the putative splice site. Sequences that aligned in the MAFFT analysis with the newly identified transcripts, showing coincident exon junctions, were assumed to have the same exon composition.

### RT-PCR amplification and sequencing of *vif* transcripts

Since no *vif* RNAs were detected in any sample by using primers recognizing sequences common to all HIV-1 DS RNAs, they were amplified with nested PCR, following RT-PCR for SS RNAs, using a *vif*-specific antisense primer (CCCTAGTGGGATGTGTACTTCTGAACTTA, HXB2 positions 5192–5220) recognizing a segment between exons 2 and 3 which, among HIV-1 spliced transcripts, is present only in *vif* RNAs. The amplified product was subjected to bulk sequencing using ABI 3730 XL automated sequencer (Thermo Fisher Scientific, Waltham, MA USA).

### PCR amplification and sequencing of proviral genome fragments

In two samples, sequences around splice site positions in the HIV-1 mid-genome portion (A1 through A5a) (which were absent in the sequenced RNAs) were obtained from proviral DNA. This was extracted from CD4^+^CD25^+^ lymphocytes using QIAamp DNA Mini Kit (Qiagen, Venlo, Netherlands) and amplified by nested PCR. Sequences and HXB2 positions of PCR primers were GGCATTCCCTACAATCCCCAAAGTC (4647–4671) and ATATGCTTTAGCATCWGATGCACA (6384–6407) in first round PCR, and AGGGGAAAGAATAATAGACATAATAGCAWCAG (4817–4848) and TCTYTCCACACAGGTACCCCATA (6342–6364) in nested PCR. PCR products were directly sequenced with ABI 3730 XL sequencer.

*vif* RNA and proviral DNA sequences were deposited in GenBank, under accessions KU901577- KU901587.

### ResultsPCR-positive samples and clinical data

In 19 of 22 samples in which HIV-1 DS RNA amplification was attempted, PCR products of expected sizes (0.2–0.9 kb) were detected through agarose gel electrophoresis (data not shown). Of the three samples with undetectable PCR products, plasma viral loads were below the level of detection (50 copies/ml) in two and it was 106 copies/ml in one. HIV-1 SS RNA amplification was attempted in 12 of the 19 samples that had yielded positive DS RNA amplification, yielding PCR products of expected sizes (0.3–1.0 kb–no product of the size expected for *vif* RNA, 1.2 kb, was observed) (data not shown). Clinical data of the 19 samples with positive RT-PCR amplification of HIV-1 spliced RNAs are shown in Table 1.

**Table 1:** Clinical data and subtypes of samples used in this study.

Sample ID	Years/months since HIV diagnosis	CD4^+^ T-cell counts (cells/μl)	Plasma viral load (copies/ml)	CDC Stage	Subtype
SPX2	11 y	219	881,000	B2	B
SPX6	9 y	243	124,550	B2	B
SPX7	4 y	986	16,000	B1	B
SPX8	8 y	585	18,800	A1	B
SPX9	14 y	385	11,400	B1	B
SPX10	3 y	475	82,600	A1	C
SPX11	4 y	910	932	A1	B
SPX12	3 y	450	35,400	A1	B
SPX13	1 y	693	78,000	A1	B
SPX15	25 y	757	2,810	C1	B
SPX18	<1 m	1042	216,000	A1	B
SPX19	10 m	871	189,000	A1	G
SPX20	5 y	534	42,600	A1	B
SPX21	3 y	622	7,430	A1	A1
SPX23	<1 m	127	12,900	C3	B
SPX24	<1 m	116	638,000	C3	B
SPX25	<1 m	9	4,150,000	C3	F1
SPX26	<1 m	237	152,000	n.a.	B
SPX35	<1 m	123	2,090,000	C3	F1

n.a.: not available

### Subtype determination

**Fig 2:**
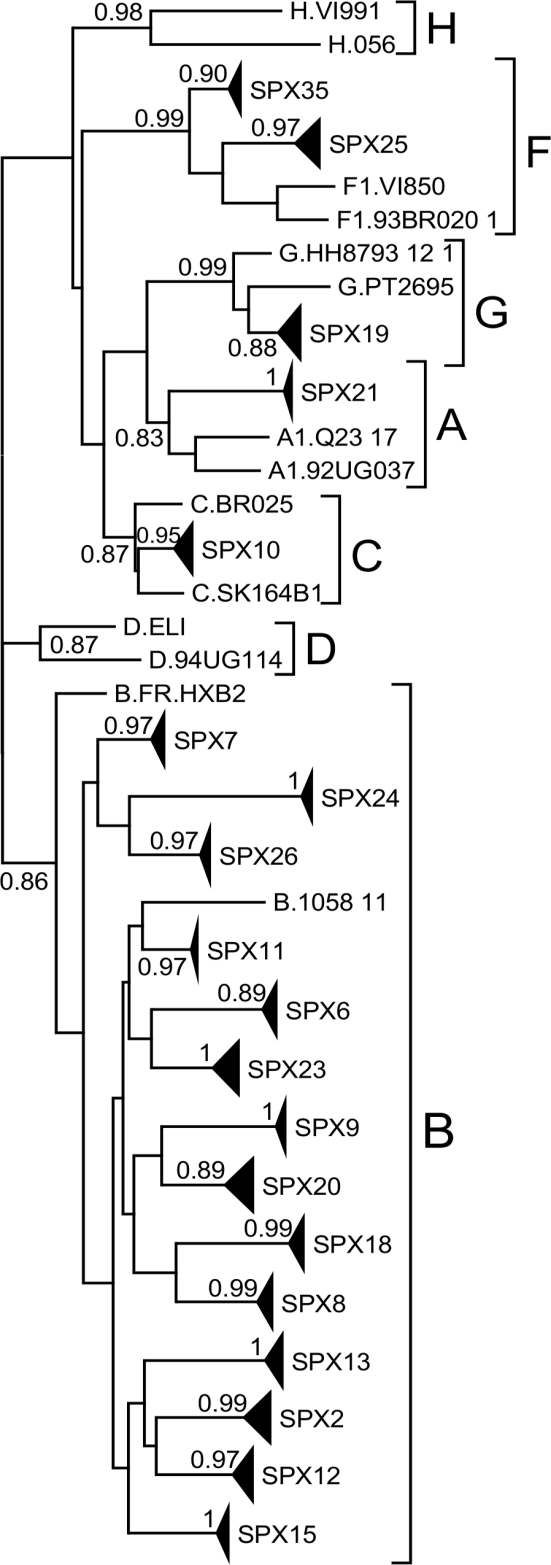
Phylogenetic tree of DS HIV-1 RNA sequences. The analysis was done with the fragment comprising exons 5 and 7 (HXB2 positions 5977–6045; 8379–8533), common to all DS transcripts, using 20 randomly chosen sequences per sample. Clades comprising sequences from each sample are compressed in triangles. SH-like node support values for sample clades and for subtype clades are shown.

A total of 13,715 reads were obtained for all samples, of which 10,199 were from the DS RNA and 3,516 from the SS RNA RT-PCR amplifications. HIV-1 subtype determination with COMET assigned 15 samples to subtype B, one (SPX10) to subtype C, one (SPX19) to subtype G, one (SPX21) to subtype A (subsubtype A1), and two (SPX25 and SPX35) to subtype F (subsubtype F1). Phylogenetic analyses of the exon 5/exon 7 fragment from 20 randomly chosen DS sequences from each sample confirmed the subtype assignation of the COMET analysis (Fig 2, Table 1).

### Number of sequences derived from HIV-1 transcripts

Using BWA-SW, 11,496 (83.8%) sequences were assigned to a single of the HIV-1 spliced transcripts used as references, while 2,029 (14.8%) sequences had ambiguous assignations (BWA-SW assigned them to more than one of the references), and 190 (1.4%) were unmappable to any of the reference transcripts. Of these, 68 corresponded to short reads 44–67 nucleotides (nt) long. Among the ambiguously assigned sequences, there were 110 in the DS RNA category assigned to both *tat* and *vpr* RNAs and 139 in the SS category assigned to *tat*, *vpr*, and *vif* RNAs. Their ambiguous assignations derived from the fact that their sequences were common to all of the assigned RNA classes, as they lacked discriminative fragments at the 5’ end which would allow assignation to a specific class. These sequences were excluded from further analyses. Another 366 sequences within the SS RNA category were ambiguously assigned to both *vpr* and *vif* RNAs, as they had sequences common to both transcripts and lacked sequences at the 5’ segment which would allow distinguishing between them. Since not a single sequence unambiguously assigned to the *vif* RNA transcript was detected in any sample, and unambiguous *vpr* RNA sequences were detected in most samples, ambiguous *vpr/vif* sequences were assigned to *vpr* RNAs. Among the ambiguously assigned sequences, there were 76 from six samples that were probable PCR-mediated artifacts. This was suspected because they lacked the usual features expected for RNA splice junctions: no known splice site was involved in the junction of discontinuous segments of the HIV-1 genome, and no GT nor AG dinucleotides were present immediately downstream of the 5’ segment and upstream of the 3’ segment, respectively, at both sides of the junction (as determined in other sequences of the corresponding sample). By alignment with reference transcripts using MAFFT and mapping sequence segments to the HXB2 genome using Sequence Locator, 2,004 ambiguous sequences could be assigned to individual HIV-1 spliced transcripts. These analyses also allowed to reassign 416 (3.6%) sequences that were incorrectly assigned by BWA-SW. There were 92 additional sequences, 7 in the assigned and 85 in the ambiguous categories, as classified by BWA-SW, that by subsequent analyses could not be unambiguously assigned to a specific HIV-1 transcript.

In total, there were 13,108 sequences which could be assigned to individual HIV-1 transcripts, of which 9,807 derived from DS RNAs and 3,301 from SS RNAs, with mean numbers per sample of 516 and 275, respectively (ranges, 203–1036 and 107–606, respectively).

### Identification of new HIV-1 splice sites

**Fig 3:**
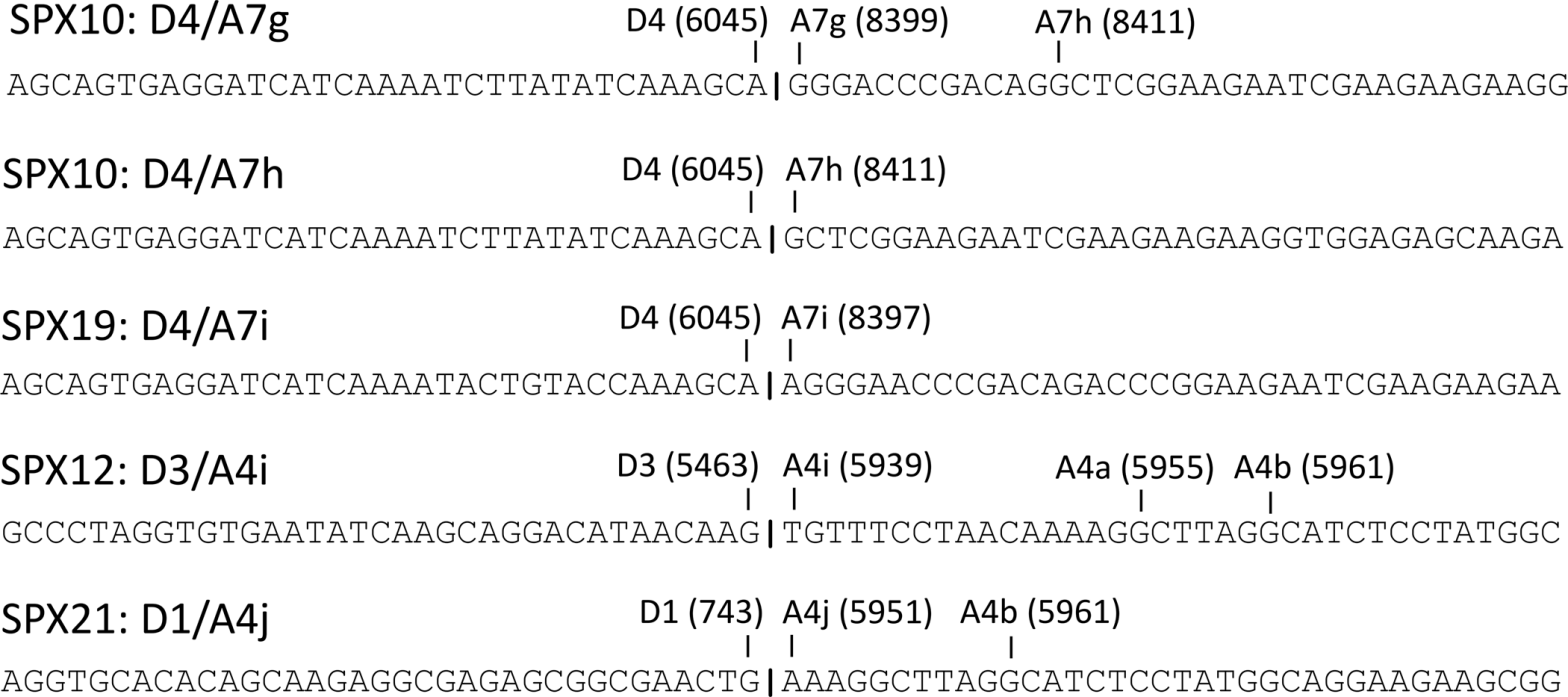
Novel splice junctions identified in this study. Consensus sequences at both sides of splice junctions are shown. Splice sites involved in splice junctions are indicated, with HXB2 positions in parentheses. Nearby splice sites used in the corresponding samples are also indicated.

In four samples, SPX10 (subtype C), SPX12 (subtype B), SPX19 (subtype G), and SPX 21 (subtype A), five 3’ss used by *nef* or *rev* RNAs mapping to unreported positions in the HIV-1 genome were identified (Fig 3).

**Fig 4:**
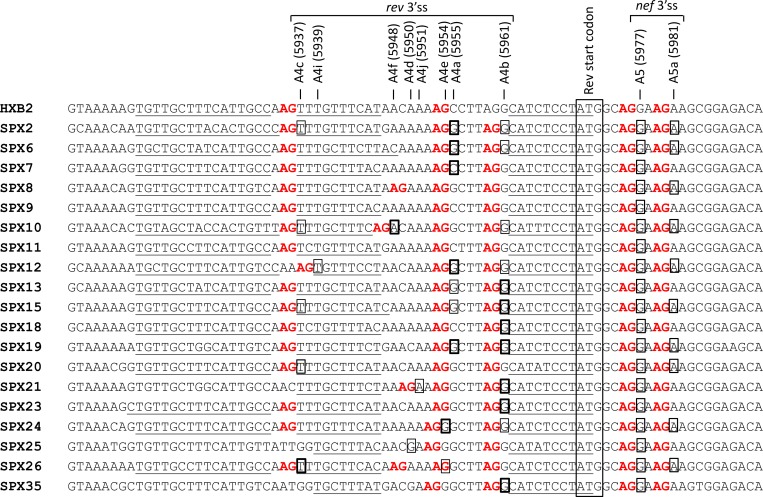
Sequences surrounding 3’ss used by *rev*, *nef* and *env-vpu* RNAs in the analyzed samples. Sequences correspond to consensuses of *tat* and/or *vpr* RNAs of the corresponding samples, except for SPX9 and SPX11 samples, in which no *tat* or *vpr* RNAs were detected, for which sequences correspond to proviral DNA. The HXB2 reference sequence is shown on top and names and positions in the HXB2 proviral genome of 3’ss used by *rev* and *nef* RNAs in this study are indicated above the alignment. AG dinucleotides immediately upstream of these sites are in red and pyrimidine-rich tracts upstream of these AGs are underlined. Nucleotides used in each sample as 3’ss are boxed, with a thicker line denoting the most commonly used *rev* 3’ss in each sample. The Rev start codon is boxed across all sequences.

**Fig 5:**
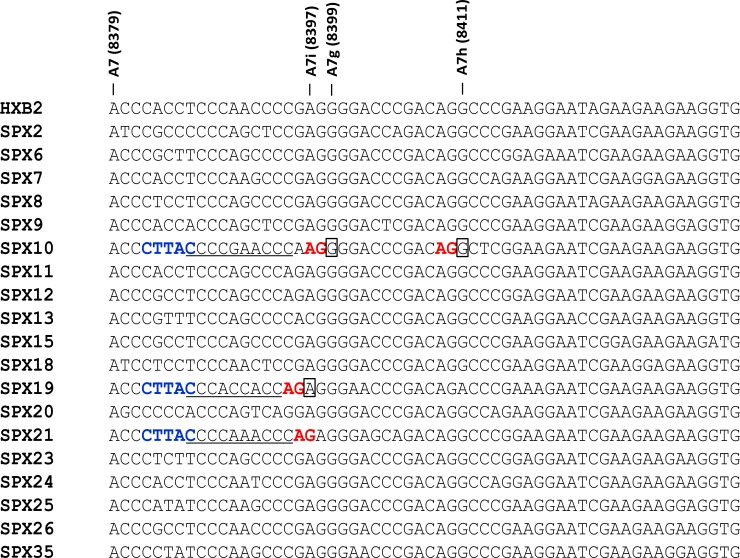
Sequences surrounding newly identified 3’ss used for splicing at the 3’-terminal exon of DS RNAs. Sequences correspond to consensuses of exon 7 of DS RNAs of the corresponding samples. The HXB2 reference sequence is shown on top and names and positions in the HXB2 proviral genome of 3’ss are indicated above the alignment. AG dinucleotides immediately upstream of these sites are in red and pyrimidine-rich tracts upstream of these AGs are underlined. Nucleotides corresponding to the new 3’ss are boxed. The yUnAy human consensus branch point sequence present upstream of the newly identified sites is shown in blue. AG, pyrimidine-rich tract, and yUnAy motif are also signaled in SPX21, although no usage of new 3’ss was detected in this sample.

In SPX10 (subtype C) some *nef* RNAs used as 3’ss for splicing at the 3’-terminal exon two 3’ss located 20 and 32 nt downstream of the usual A7 site (at HXB2 positions 8399 and 8411, respectively). These splice sites were designated A7g and A7h, respectively, and RNAs using them had exon compositions 1.5.7g, 1.3.5.7g, 1.2.3.5.7g, 1.5.7h, and 1.3.5.7h. In SPX19 (subtype G), some *nef* RNAs and a single *rev* RNA-derived sequence used for splicing at the 3’-terminal exon a 3’ss located 18 nt downstream of A7 (at HXB2 position 8397) which was designated A7i. RNAs using A7i had exon compositions 1.2.5.7i and 1.4a.7i. In SPX12 (subtype B), some *rev* RNAs used for splicing at the first coding exon a 3’ss located 16 nt upstream of A4a (at HXB2 position 5939) which was designated A4i. All RNAs using A4i had exon composition 1.3.4i.7. In SPX21 (subtype A), some *rev* RNAs used for splicing at the first coding exon a 3’ss located 4 nt upstream of A4a (at HXB2 position 5951), which was designated A4j. All transcripts using A4j had exon composition 1.4j.7. That the newly identified junctions represent real HIV-1 RNA splice junctions and are not PCR-mediated artifacts was inferred from the facts that in all of them a known HIV-1 5’ splice site (5’ss) was involved (Fig 3) and that the usual features of the metazoan 3’ss, i.e. an AG immediately upstream of the 3’ss and a pyrimidine-rich tract located further upstream, were present in the sequences of the corresponding viruses (Fig 4, Fig 5).

It should be noted that A4j is located only one nt downstream of the previously identified A4d site [11,13], and, therefore, both splice sites are mutually incompatible and could be viewed as variants of the same splice site. This incompatibility is similar to that existing between A4e and A4a, the first located only one nt upstream of the second [11]. Similarly, A4i could be viewed as a variant of A4c, located two nt upstream, since, in the hypothetical case that both sites would be present in a virus, A4i would necessarily be preceded by GAG, which is associated with inefficient cleavage [65] resulting in a most likely nonfunctional site, and, consequently, to mutual incompatibility of A4c and A4i. A similar consideration could be made regarding A7g and A7i, separated by two nt.

Of the newly identified 3’ss or variants of 3’ss, A7g, A7h, and A7i were used by small minorities of *nef* transcripts (S2 Table), but A4i and A4j were used by substantial proportions of *rev* RNAs in SPX12 and SPX21 viruses, respectively (see below).

A4i and A4j are located upstream of the coding sequence for Rev and their usage does not result in the modification or the creation of any open reading frame (Fig 4). A7i, A7g and A7h are located upstream of the Nef coding sequence and their usage would not affect the coding potential of Nef RNAs. However, in RNAs using 3’ss of the first coding exons of *tat* and *rev* RNAs, usage of A7g and A7h would result in a frameshift and usage of A7i would result in six amino acid deletions in the second coding exons of Rev and Tat proteins. It must be noted, however, that all but one sequences using A7g, A7h and A7i correspond to *nef* RNAs, the only exception being a *rev* RNA using A7i (S2 Table).

No usage of novel 5’ splice sites was detected in any of the transcripts.

### Relative abundance of sequences derived from HIV-1 DS and SS RNAs according to encoded proteins

#### DS RNAs

**Fig 6:**
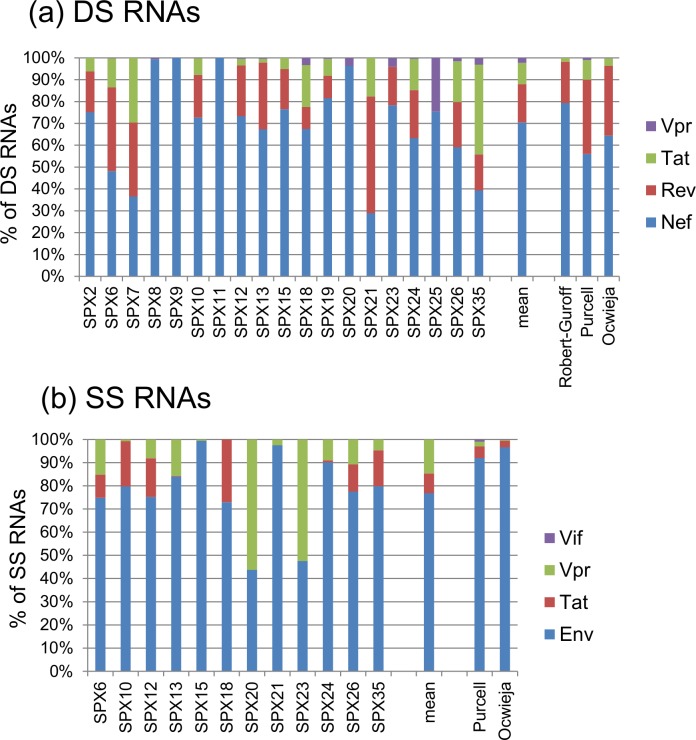
**Relative abundance of RNAs coding for different proteins among (a) DS and (b) SS HIV-1 RNAs.** The vertical axis represents proportions relative to all transcripts of each category. Mean proportions of all samples are shown on the right of the columns for individual samples. For comparison, proportions reported in *in vitro* studies by Robert-Guroff et al. [2], Purcell and Martin [6], and Ocwieja et al. [9] are shown.

DS transcripts potentially coding for Nef, Rev, Tat, and Vpr proteins, according to the splice sites used, were detected in 19, 14, 13, and 13 samples, respectively (Fig 6A, S2 Table). In 17 samples, *nef* RNAs were the most abundant, in agreement with previous *in vitro* studies [1,2,6,9]. However, in two samples (SPX21, of A subtype, and SPX35, of F subtype) *rev* and *tat* RNAs, respectively, were more abundant than *nef* RNAs (Fig 6A). On average, *nef* RNAs represented around 70% DS RNAs, followed, in order of abundance, by *rev*, *tat* and *vpr* RNAs.

#### SS RNAs

With the exclusion of ambiguous *tat/vpr/vif* sequences and the assumed *vpr* assignation of ambiguous *vif/vpr* sequences, as discussed above, *env-vpu*, *tat*, and *vpr* RNAs were detected in 12, 8, and 10 samples, respectively. No unambiguous *vif* RNAs were detected in any sample by using primers recognizing sequences common to all SS RNAs and pyrosequencing. However, by using a *vif* RNA-specific primer in the nested PCR and Sanger sequencing, *vif* RNAs were detected in nine samples: SPX6, SPX10, SPX12, SPX13, SPX15, SPX20, SPX23, SPX24, and SPX35 (data not shown). On average, *env-vpu* RNAs were the most abundant, representing around 77% SS RNAs, followed, in order of abundance, by *vpr* and *tat* RNAs (Fig 6B, S3 Table).

### Incorporation of extra small noncoding exons

#### Incorporation of exons 2 or 3

**Fig 7:**
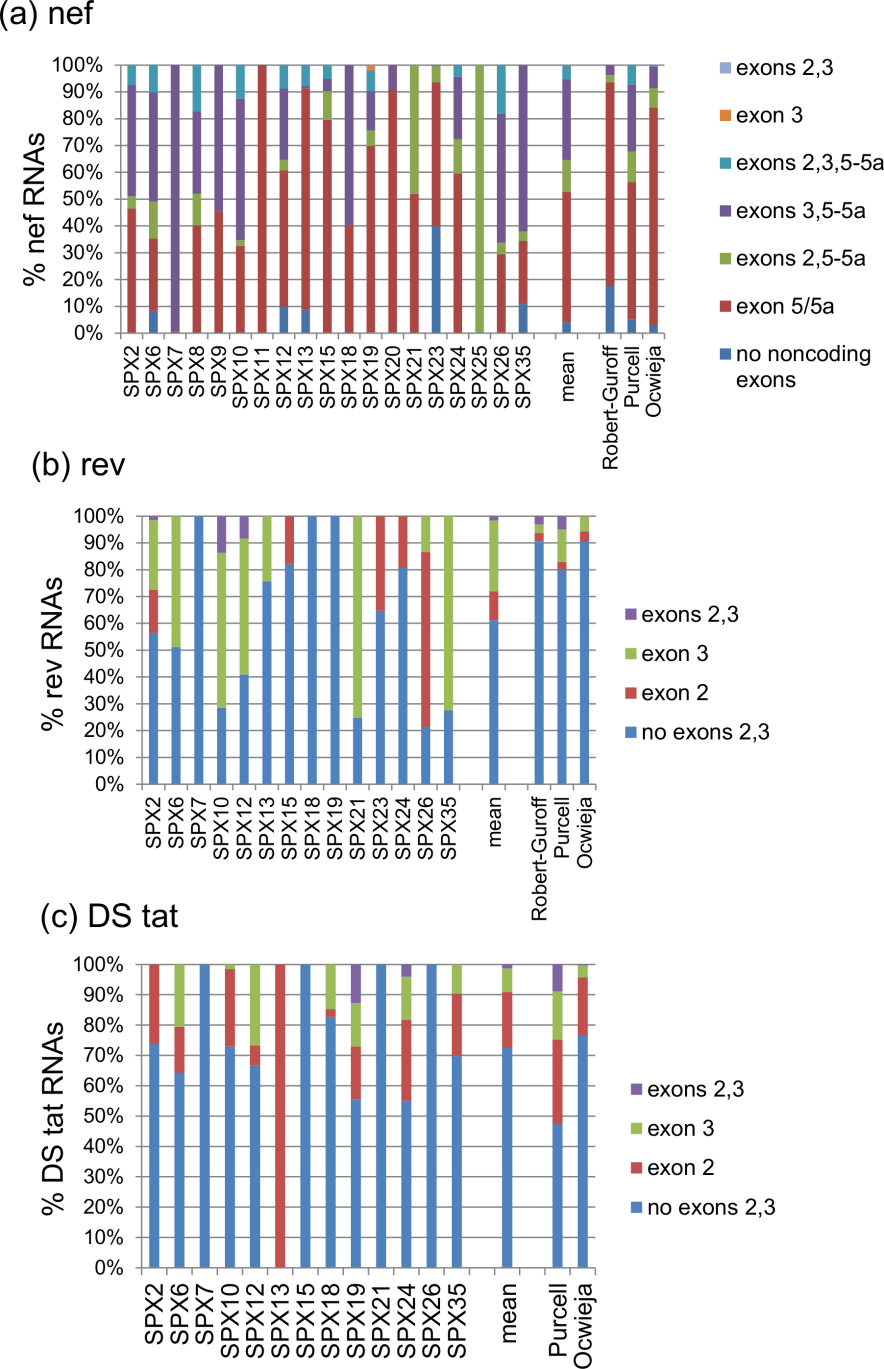
**Frequency of incorporation of noncoding exons in DS RNAs among (a) *nef*, (b) *rev*, and (c) *tat* RNAs.** The vertical axis represents proportions relative to all transcripts of each class. Mean proportions of all samples are shown on the right of the columns for individual samples. For comparison, proportions reported in *in vitro* studies by Robert-Guroff et al. [2], Purcell and Martin [6], and Ocwieja et al. [9] are shown.

**Fig 8:**
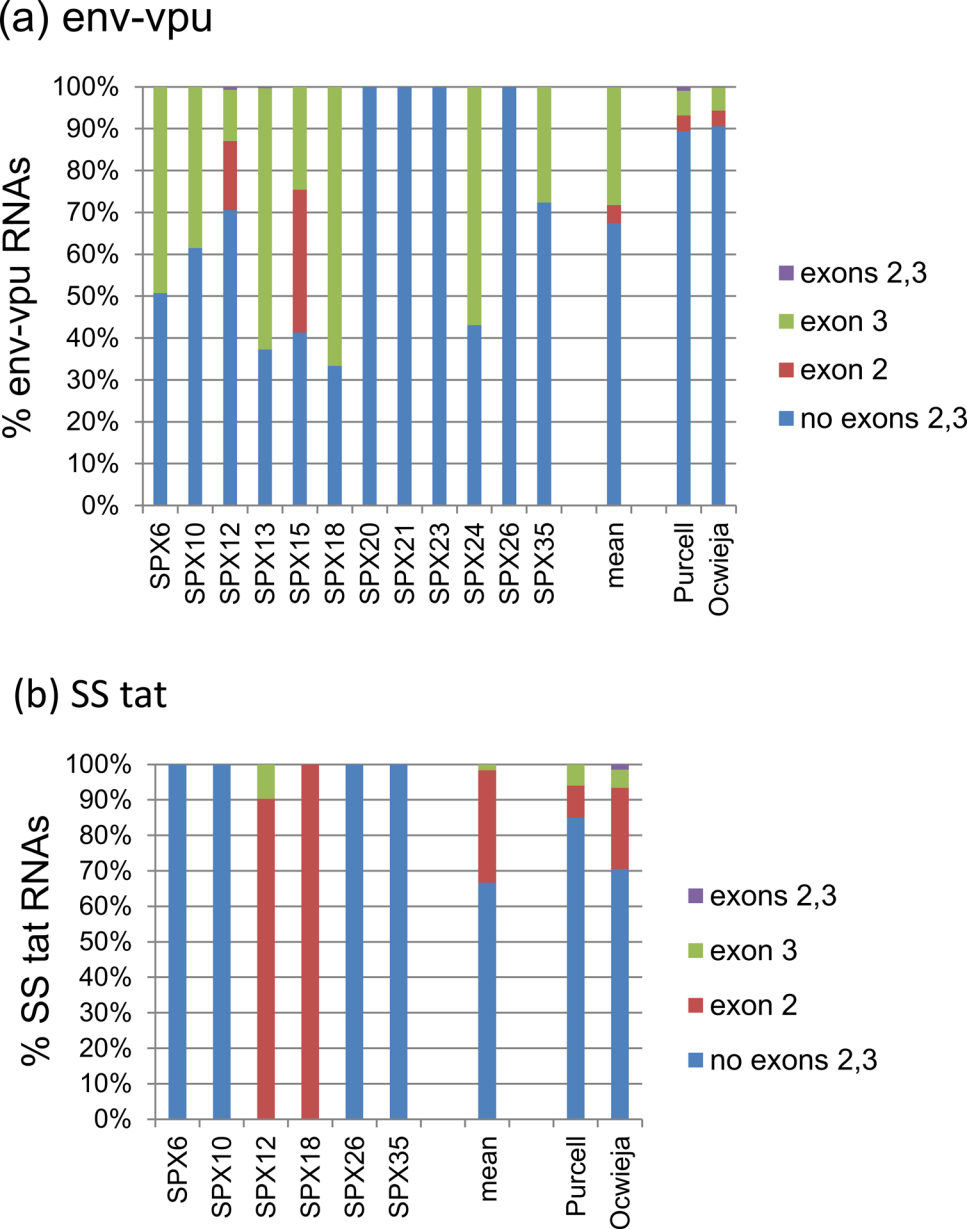
**Frequency of incorporation of noncoding exons in SS RNAs among (a) *env-vpu* and (b) *tat* RNAs.** The vertical axis represents proportions relative to all transcripts of each class. Mean proportions of all samples are shown on the right of the columns for individual samples. For comparison, proportions reported in in vitro studies by Purcell and Martin [6], and Ocwieja et al. [9] are shown.

The frequencies of incorporation of exons 2 or 3 or both into *nef*, *rev*, and DS *tat* RNAs are shown in Fig 7, and those corresponding to *env-vpu* and SS *tat* RNAs are shown in Fig 8.

*nef* RNAs incorporating exons 2 or 3 were detected in 18 samples, and in 10 samples, *nef* transcripts incorporating one or both of these exons were the most abundant. Among samples expressing *nef* RNAs incorporating exons 2 or 3, inclusion of exon 3 was more frequent in 13 and that of exon 2 in 4.

Among *rev* RNAs, in 11 of 14 samples in which they were expressed, transcripts incorporating exons 2 or 3 were identified, and in 5 they were more abundant than transcripts lacking them. Similarly to *nef* RNAs, there was a preference for inclusion of exon 3, with 8 of 11 samples in which extra noncoding exons were detected expressing more abundantly transcripts incorporating exon 3.

With regard to DS *tat* RNAs, they incorporated exons 2 or 3 in 9 of 13 samples in which these RNAs was expressed. In contrast to *nef* and *rev* RNAs, there was an overall preference for inclusion of exon 2 over exon 3, with 6 of 9 samples showing higher abundance of RNAs including exon 2. Preference for inclusion of exon 2 was also observed among SS *tat* RNAs, which was seen in both samples in which extra noncoding exons were incorporated into these RNAs.

Among *env-vpu* RNAs, extra noncoding exons were incorporated in 7 of 12 samples, with a preference for inclusion of exon 3 (7 samples) over exon 2 (2 samples).

With regard to *vpr* RNAs, incorporation of exon 2 was seen in a single DS sequence of one sample.

#### Nef transcripts lacking noncoding exon 5

Most *nef* RNAs incorporate noncoding exon 5 [1,2,6], but it has been reported that a minority of *nef* RNAs can be generated through direct splicing from 5’ss D1 to 3’ss A7 (*nef* 1.7 RNAs), thus excluding exon 5 [2,6,9,35]. We found *nef* 1.7 RNAs in 6 samples, in which they represented a minority of *nef* RNAs, although in SPX23 they represented 40% *nef* transcripts. In one sample (SPX19, subtype G), two other *nef* RNA species lacking exon 5, with exon compositions 1.3.7 and 1.2.3.7, were detected at very low proportions.

### Alternative 3’ss usage

#### Alternative 3’ss usage by *rev* RNAs (Fig 9A, S2 Table

**Fig 9:**
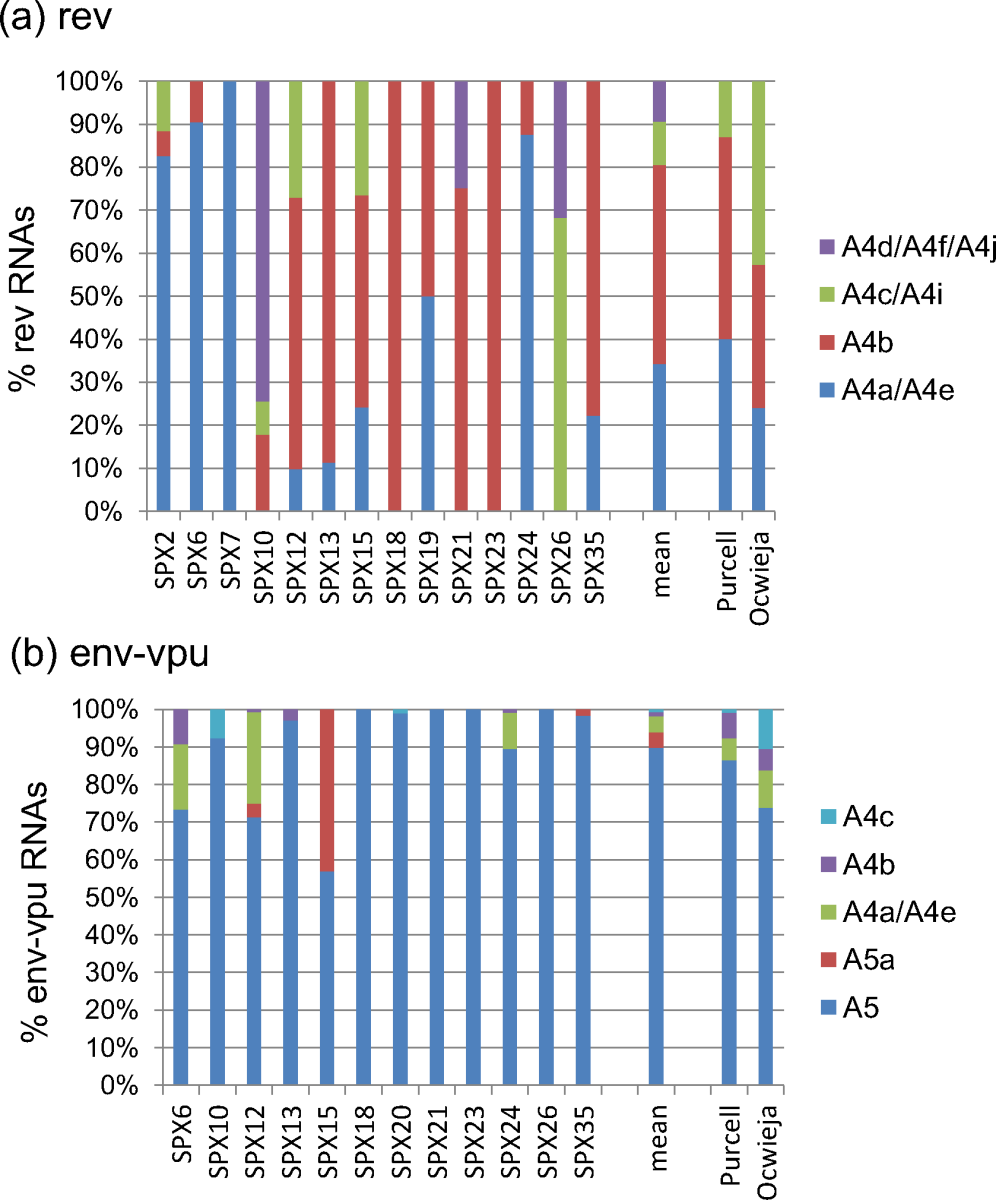
**Alternative 3’ss usage by (a) *rev* and (b) *env-vpu* RNAs.** 3’ss separated by one or two nt (A4a/A4e, A4c/A4i, A4d/A4f/A4j) were grouped together. A4e is used by SPX24 and SPX35, A4d by SPX26, A4f by SPX10, A4j by SPX21, and A4i by SPX12. The vertical axis represents proportions of RNAs using each 3’ss relative to all transcripts of each class. Mean proportions of all samples are shown on the right of the columns for individual samples. For comparison, proportions reported in *in vitro* studies by Purcell and Martin [6] and Ocwieja et al. [9] are shown.

Eight different 3’ss were used for splicing at the first coding exon of *rev* RNAs, A4a, A4b, A4c, A4d, A4e, A4f, A4i, and 4j (the number would be four if sites separated by one or two nt, A4a/A4e, A4c/4i, and A4d/A4f/A4j, would be grouped as variants of the same 3’ss). The proportions of *rev* RNAs in which usage of these 3’ss were detected are shown in Fig 9A, in which splice sites separated by one or two nt are grouped together, for the reasons explained above. A4a/A4e was preferred in 4 samples; A4b in 7; A4cand A4f each in one sample; and almost equal proportions of A4a- and A4b-using RNAs were found in one sample. With regard to *rev* RNAs using unusual or novel 3’ss or variants of 3’ss (A4d, A4e, A4f, A4i and A4j) their abundance was in all cases greater than 20% of total *rev* transcripts in the samples in which they were detected. In two samples, of subtypes B and C, A4e and A4f, were, respectively, the most commonly used 3’ss for *rev* RNA generation.

#### Usage of 3’ss A5a by *nef* and *env-vpu* RNAs (Fig 9B, S2 and S3 Tables)

3’ss A5 is used for generation of most *nef* and *env-vpu* RNAs [1–3,5,6]. However, an alternative 3’ss, A5a, located 4 nt downstream of A5 (at HXB2 position 5981) has been reported to be used by some *nef* [8,9] and *env-vpu* [8,9,16] RNAs. Among the analyzed samples, A5a was used by *nef* RNAs in 7 samples and by *env-vpu* RNAs in 3 samples. Surprisingly, there was no coincidence in A5a usage between *env-vpu* and *nef* RNAs, so that, in total, A5a was used by *nef* or *env-vpu* RNAs in 10 samples. The proportion of *nef* RNAs using A5a was less than 10% in all cases. Notably, transcripts using A5a represented 43% of *env-vpu* RNAs in one sample (SPX15).

#### Alternative 3’ss usage in the 3’-terminal exon of DS RNAs (S2 Table)

In most HIV-1 DS RNAs, 3’ss A7 is used for splicing at the 3’-terminal exon [1,2,6]. However, occasional usage of 3’ss A7a and A7b, located 24 and 28 nt, respectively, upstream of A7 (at HXB2 positions 8355 and 8351, respectively), has been reported in HXB2 [1] and p89.6 [9] isolates. We detected usage of A7b or both A7a and A7b in 4 samples. Additionally, in two samples, we detected usage of three unreported 3’ss for splicing at the 3’-terminal exon, which were designated A7g, A7h, and A7i. The number of samples in which the alternative 3’ss were used was five: A7a and A7b in SPX2 and SPX24 (subtype B), A7b in SPX8 (subtype B), A7g and A7h in SPX10 (subtype C), and A7b and A7i in SPX19 (subtype G). The alternative sites were used by *nef* RNAs in all five samples, by *rev* RNAs in two, and by *tat* RNAs in two. The proportions of RNAs using these sites were in each case less than 5% of *nef*, *rev*, and *tat* RNAs. Regarding A7g, A7h, and A7i, each was used in around 2% of *nef* RNAs in samples in which they were detected. In addition, usage of A7i was detected in 1 of 88 *rev* RNAs in SPX19.

#### Alternative 3’ss usage by *env-vpu* RNAs (Fig 9B, S3 Table)

Six different 3’ss were used for splicing at the *env-vpu* coding exon: A5, A5a, A4a, A4b, A4c, and A4e (the number would be five if A4e would be considered a variant of adjacent A4a). The proportions of *env-vpu* RNAs in which usage of these 3’ss were detected are shown in Fig 9B. A5 was preferred in all *env-vpu* RNAs, with exclusive A5 usage in five, although, as stated above, in SPX15 almost half *env-vpu* RNAs used A5a. Surprisingly, in the subtype C sample SPX10, no *env-vpu* RNAs splicing at A4f (the 3’ss preferentially used by *rev* RNAs in this sample) were detected, with A4c being the second 3’ss used by *env-vpu* RNAs after A5.

## Discussion

This study provides new data on *in vivo* HIV-1 RNA splice site usage in viruses of different subtypes (A, B, C, F, and G), for which previously available data were scarce. A high diversity of spliced transcripts were identified through pyrosequencing, several of which involve usage of five previously unreported splice sites (Fig 3, Fig 4, Fig 5), four of them identified in non-B subtypes. The new splice sites correspond to 3’ss used by *nef* or *rev* RNAs. Although the usage of the three new sites at the 3’-terminal exon of DS RNAs (designated A7g, A7h, and A7i) was uncommon, usage of both newly identified 3’ss for splicing at the first *rev* coding exon, named A4i and A4j, was relatively frequent (Fig 9A). Usage of the new 3’ss was associated with the presence of AG dinucleotides immediately upstream of the splice sites and a pyrimidine-rich tract further upstream (Fig 4, Fig 5). As explained above, A4i and A4j map to positions in the HIV-1 genome located just one or two nt from previously reported A4c and A4d sites, respectively, and, therefore, they could be viewed as variants of these sites rather than new splice sites. Similarly, A7g and A7i, separated by two nt, could be viewed as variants of the same splice site. It should be pointed out, however, that reported HIV-1 splice sites separated by one or two nt, such as A4a and A4e, or A4d and A4f, have been considered and named as distinct splice sites [11,12]. In accordance with the scanning model of 3’ss recognition by the splicing machinery [65,66], the nucleotide after the first AG encountered downstream of the branch point is used as 3’ss, unless another downstream AG is located in close proximity, which can compete for 3’ss recognition. The competitiveness of the AG varies according to the nucleotide preceding it, following this order: CAG ≈ UAG > AAG > GAG [66]. This model is consistent with the usage of novel and unusual 3’ss by *rev* RNAs observed in our study (Fig 4). Thus, we see that in SPX10, the A4b site, preceded by UAG, is used in 17.7% of *rev* RNAs, even though upstream of it there is the A4f site, preceded by CAG; and that A4b, with preceding UAG, is used preferentially to the upstream 3’ splice sites A4j in SPX21 and A4e in SPX35, respectively, which are preceded by AAG. However, the proposed hierarchy of 3’ss usage is not followed in SPX24. In this virus, the upstream site, A4e, with preceding AAG, is preferentially used over A4b, with preceding UAG, a preference also observed in *env-vpu* RNAs. Among the unusual 3’ss, we observed the preferential usage of A4f by *rev* RNAs of a subtype C virus (SPX10), confirming a previous observation in subtype C viruses in an *in vitro* infection assay [12]. However, in contrast to the previous study, A4b was used by a substantial proportion of *rev* transcripts of SPX10. With A4i and A4j sites, the number of 3’ss mapping to different positions in the HIV-1 genome reported to be used by *rev* RNAs is ten. The great multiplicity of splice sites used by *rev* transcripts may derive from the great variability of the Tat coding sequence [67], where *rev* 3’ss are located. Mutations in this region may give rise to new AG dinucleotides, generating new 3’ss recognized by the splicing machinery scanning downstream of a branch point.

With regard to the newly identified 3’ss used for splicing at the 3’-terminal exon of DS transcripts (A7g, A7h, A7i), the presence of the upstream AG and a pyrimidine-rich tract may not explain by themselves their usage in SPX10 and SPX19, since other viruses here analyzed have these sequence features in the same genome region (Fig 5). The difference in splice site usage may be explained by the fact that SPX10 and SPX19 have the human consensus branch point sequence yUnAy [68] (with the lower case denoting less conserved nucleotides) upstream of the A7g/A7h/A7i sites, which is absent from the other viruses, except the subtype A virus SPX21 (Fig 5). In this virus, sequence features (yUnAy motif, pyrimidine-rich tract, AG dinucleotide) could also predict usage of a 3’ss at the nt position between A7g and A7h; failure to detect it could be explained by the relatively low number of *nef* RNA sequences, among which usage of all but one of the new sites close to A7 was detected, obtained for SPX21, compared to SPX10 or SPX19, and by the low frequency of usage of these sites. Although the pyrimidine-rich tracts located between the proposed branch points and the 3’ss in SPX10 and SPX19 have only seven pyrimidines interspersed with purines, there are reported 3’ss for which the pyrimidine contents of the upstream sequences were much lower than those seen for A7g, A7h, and A7i [69,70]. Failure to detect usage of A7h in SPX19, in spite of having the same yUnAy and adjacent AG motifs as SPX10 (Fig 5), may derive from the fact that the CAG sequence upstream of A7i in SPX19 could make it a stronger 3’ss, compared to A7g in SPX10, which is preceded by AAG, thus preventing usage of the potential 3’ss located 14 nt downstream [66].

Other unusual splice sites detected in this study were A5a, A7a, and A7b. A5a, located 4 nt downstream of A5, was previously identified in a small minority of *env* transcripts in two isolates [9,18], and *in vivo* in one of five viruses [8]. In the present study, usage of A5a was detected in 10 (53%) samples. *nef* or *env*-*vpu* RNAs using A5a were a small minority relative to those using A5, except in one sample in which 43% of *env-vpu* transcripts used A5a (Fig 9B). No unusual sequence feature was found that would explain the high frequency of A5a usage by *env-vpu* RNAs in this sample, as the sequence surrounding A5 and A5a was CAGGAAGA (with the underline denoting the splice sites), which is highly conserved in group M viruses. Usage of A7a or A7b, previously reported only in the HXB2 [1] and p89.6 [9] isolates, was detected in three samples, mostly in *nef*, but also in *tat* and *rev* RNAs, representing a small minority within each RNA class.

In addition to the new and unusual HIV-1 transcripts identified in this study, a mention should also be made to previously reported unusual HIV-1 transcripts that were not detected. These include RNAs containing exon 6D, delimited by 3’ss A6 and 5’ss D5, coding for the chimeric protein Tev/Tnv [1,14,15], or using splice sites D2a [12], A4g [12], A4h [13], A7c [17], A1A [18], D1A [18], or cryptic splice sites whose usage was detected through deep sequencing in a very small minority of transcripts in the p89.6 isolate [9]. Other 3’ss used by short RNAs splicing near the HIV-1 genome’s 3’ end [7–9] were not detected in our assay because the primers were not designed for their amplification. These RNAs were not the subject of the current study, due to their reported relatively infrequent detection *in vivo* and their still undefined role in HIV-1 life cycle. Future studies will need to be carried out to better define their abundance, splice site usage and function.

Usage of splice sites reported to be commonly used by HIV-1 isolates failed to be detected in some samples (S2 and S3 Tables): 3’ss for *rev* RNA in four samples, A1 in three samples, A2 in one sample, and A3 in six samples. Sequence analysis around these 3’ss revealed the presence of the usual upstream AG dinucleotide and pyrimidine-rich tracts (Fig 4, S1 Fig). Therefore, lack of detection of RNAs using these splice sites in some samples may not be caused by mutations in their essential elements, but rather by expression levels below the level of detection of our assay.

This study was the first to analyze splicing patterns in viruses of HIV-1 subtypes A, F, and G, and *in vivo* splicing patterns in a subtype C virus. In these viruses, splice site usage was largely consistent with that seen in subtype B viruses, but we note that four of the five newly identified splice sites or splice site variants were found in non-subtype B viruses. One of these was A4j, used by some *rev* RNAs in the subtype A1 virus SPX21. However the AG dinucleotide adjacent to this site is found in none of the available subtype A1 near full-length genomes sequences [71], and, therefore, A4j usage would probably be rare in subtype A viruses. In the subtype C virus, preferential A4f usage by *rev* RNAs, previously reported in two of three isolates [12], was confirmed. A4f usage is predicted in most subtype C viruses, considering that the AG dinucleotide immediately upstream is present in most of them (S2 Fig). Usage of A4e (which could be viewed as a variant of A4a shifted one nt upstream) detected in the subtype F virus SPX35, is also predictable in the great majority of F1 subtype viruses, considering that the AG dinucleotide upstream of A4e is present in all available subtype F1 near full-length genome sequences (for the same reason, A4e would also be predicted to be used by most CRF02_AG viruses) (S2 Fig). With regard to the newly identified 3’ss used for splicing at the 3’-terminal exon, the yUnAy motif and downstream pyrimidine-rich and CAG sequences present in the consensus sequences of subtypes C, F1 and G and of CRF02_AG could predict usage of A7h in most viruses of subtypes C and F1, and of A7i in most viruses of subtype G and of CRF02_AG (S3 Fig).

Many of the quantitative data on relative expression of HIV-1 RNAs obtained in this study are consistent with previously published data [1,2,6,8,9]. These include (1) the greater relative abundance of *nef* and *env-vpu* RNAs among DS and SS transcripts, respectively, in most samples; (2) the order of relative abundance *nef>rev>tat>vpr* among DS RNAs in a majority of samples; (3) the preferential incorporation of exon 3 in *nef*, *rev*, and *env-vpu* RNAs, and of exon 2 in *tat* RNAs; (4) the relative rarity of *nef* 1.7 RNAs compared to *nef* RNAs incorporating exon 5; and (5) the infrequent use of A5a or of A7a/A7b, compared to A5 and A7, respectively. However, there are other data that differ from previous reports. One of these is the substantially greater relative abundance of *vpr* RNAs among SS RNAs (mean 14.7%, vs. 8.5% for SS *tat* RNAs), with 7 of 12 samples showing greater SS *vpr* RNA-derived than SS *tat* RNA-derived PCR products. Greater SS *vpr* than SS *tat* RNA expression has also been observed by us in an *in vitro* acute infection assay of PBMCs by HIV-1 primary isolates (unpublished data). The much lower relative abundance of DS *vpr* detected by us (mean 2.3% of DS RNAs) could be explained by nonsense-mediated RNA decay of DS *vpr* transcripts, which contain a stop codon more than 50–55 nt upstream of an exon-exon junction [72], and by the fact that DS *vpr* RNAs frequently undergo rapid splicing to *nef*, *rev* and *tat* RNAs [4]. For the interpretation of the quantitative data, it may be important to take into consideration the possible existence of biases derived from (1) unequal amplification efficiencies of templates of different lengths coamplified in the same reaction; and (2) the relatively low abundance of HIV-1 spliced transcripts in lymphocytes from HIV-1-infected individuals compared with *in vitro*-cultured cells. With regard to the length-dependent bias, it is shared with other PCR-based assays employed in previous studies in which relative quantifications of spliced RNAs were reported [2,6,9]. The relatively low abundance of HIV-1 spliced transcripts in lymphocytes from HIV-1-infected individuals could explain the failure to detect in some samples less abundant transcripts (i.e., those different from *nef* or *env-vpu* RNAs among DS and SS RNAs, respectively), which might be below the detection threshold in our assay. Even with these methodological caveats in mind, considering the agreement of many of our quantitative data with previously published data, those differing from them, particularly those observed in a majority of samples, should not be dismissed, pending confirmation by other authors and with different assays.

Since in this study *in vivo* expression of HIV-1 spliced RNAs was examined, it should be considered that in this setting there are nonviral factors that could influence HIV-1 splice site usage, including host genetic factors and disease stage. These are out of the intended scope of the present study and were not analyzed.

In conclusion, in this study, in which we examine *in vivo* expression of individual HIV-1 spliced transcripts through pyrosequencing, using samples corresponding to viruses of five different subtypes, we identified five new splice sites or splice site variants used by HIV-1 *nef* and *rev* RNAs. Four of these were detected in viruses of non-B subtypes, which anticipates finding greater variability in HIV-1 RNA splice site usage by analyzing larger number of viruses of different genetic forms. Further work will be required to obtain mechanistic insights into the regulation of the usage of the newly identified splice sites through the action of nearby enhancer or silencer motifs or through RNA secondary structures [31,32,73]. Many data on relative expression among the HIV-1 spliced RNA categories were consistent with previous reports based on *in vitro* assays, but some were different, which may need to be confirmed in future studies. Since RNA splicing is an essential step in the HIV-1 life cycle, the results here reported may be of relevance for the development of therapeutic agents aimed at interfering with HIV-1 splicing mechanisms, some of which have shown promising results in *in vitro* assays [74–77].

## Supporting Information

S1 Fig Sequences surrounding A1, A2, and A3 3’ss in samples in which their usage was not detected. AG dinucleotides immediately upstream of these sites are in red and pyrimidine-rich tracts upstream of these AGs are underlined.
(TIFF)

S2 Fig Consensus sequences of most globally prevalent HIV-1 clades around 3’ss of *rev* and *nef* RNAs. Names and HXB2 positions of 3’ss used by *rev* and *nef* RNAs in this study are indicated above the alignment. AG dinucleotides potentially used as 3’ss are in red and pyrimidine-rich tracts upstream of these AGs are underlined, The Rev start codon is boxed across all sequences.
(TIF)

S3 Fig Consensus sequences of most globally prevalent HIV-1 clades at the 5’ segment of exon 7. Names and HXB2 positions of 3’ss newly identified this study (A7g, A7h, A7i) are indicated above the alignment. AG dinucleotides adjacent to potentially used A7h and A7i sites, according to the presence of and adjacent upstream A, C or T, and further upstream pyrimidine-rich tract (underlined) and yUnAy motif (in blue), are in red.
(TIF)

S1 Table Data associated with sequence files submitted to the European Nucleotide Archive. (XLSX)

S2 Table Distribution of HIV-1 doubly spliced RNAs identified in each sample. (XLSX)

S3 Table Distribution of HIV-1 singly spliced RNAs identified in each sample. (XLSX)
