# Supplementary figures and images for: Sequence Analysis of In Vivo-Expressed HIV-1 Spliced RNAs Reveals the Usage of New and Unusual Splice Sites by Viruses of Different Subtypes
Source: PLoS One. 2016 Jun 29;11(6):e0158525. doi: 10.1371/journal.pone.0158525 (PMC4927154; doi:10.1371/journal.pone.0158525)

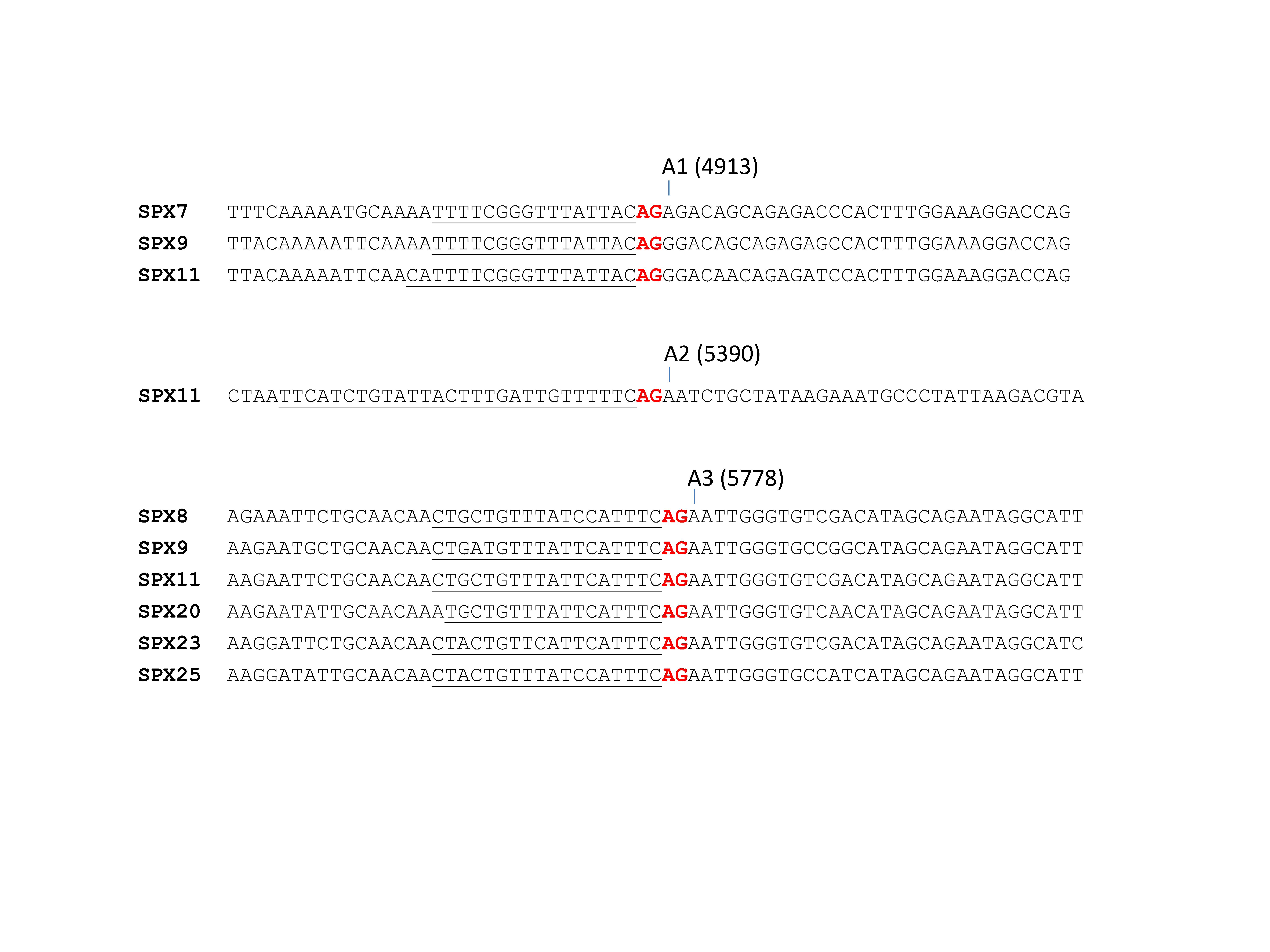

Supplement: S1 Fig — AG dinucleotides immediately upstream of these sites are in red and pyrimidine-rich tracts upstream of these AGs are underlined. (TIFF) [file pone.0158525.s001.tiff]

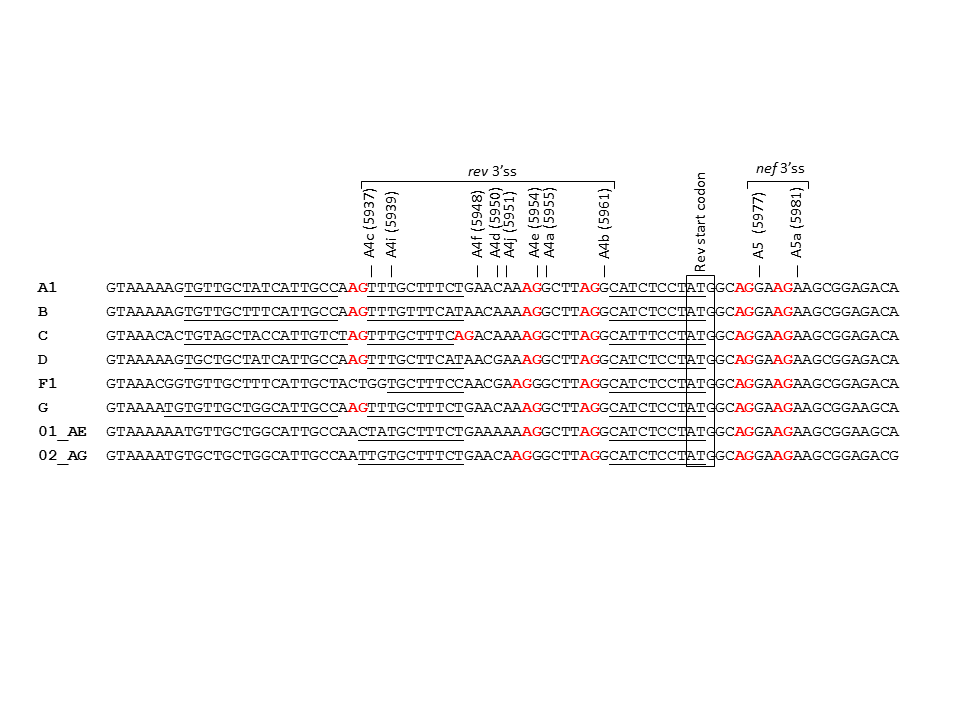

Supplement: S2 Fig — Names and HXB2 positions of 3’ss used by rev and nef RNAs in this study are indicated above the alignment. AG dinucleotides potentially used as 3’ss are in red and pyrimidine-rich tracts upstream of these AGs are underlined, The Rev start codon is boxed across all sequences. (TIF) [file pone.0158525.s002.tif]

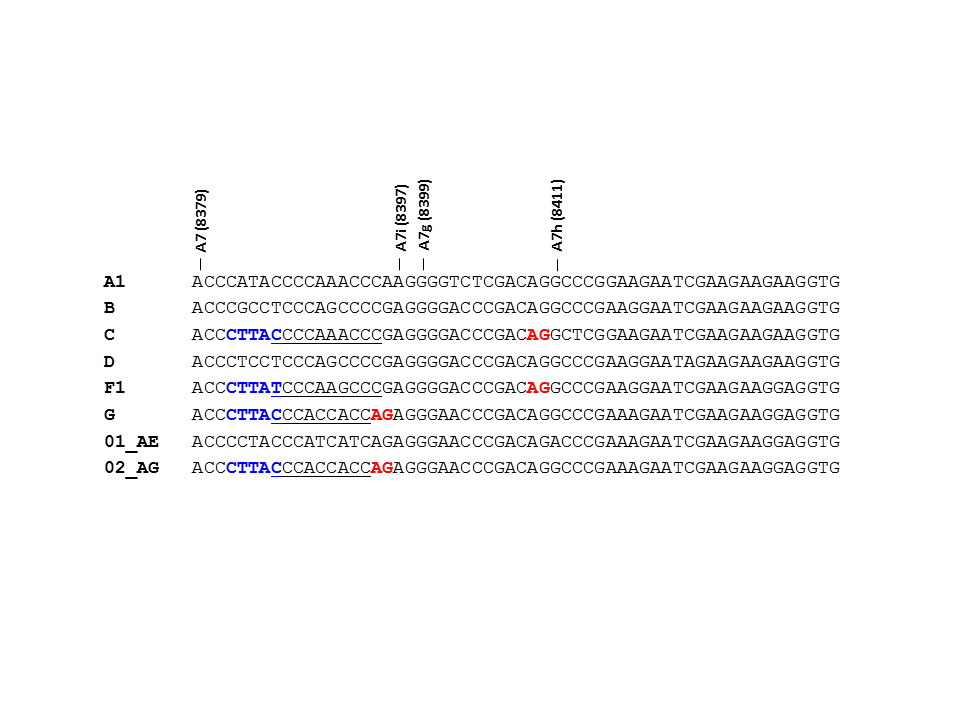

Supplement: S3 Fig — Names and HXB2 positions of 3’ss newly identified this study (A7g, A7h, A7i) are indicated above the alignment. AG dinucleotides adjacent to potentially used A7h and A7i sites, according to the presence of and adjacent upstream A, C or T, and further upstream pyrimidine-rich tract (underlined) and yUnAy motif (in blue), are in red. (TIF) [file pone.0158525.s003.tif]
